# The Beyond Reality Image Collection (BRIC)

**DOI:** 10.3758/s13428-024-02586-y

**Published:** 2025-01-07

**Authors:** Noga Segal-Gordon, Yoav Bar-Anan

**Affiliations:** School of Psychological Sciences, Tel Aviv University, Tel Aviv, Israel

**Keywords:** Evaluative ratings, Affective rating, Visual stimuli, Images, Norms

## Abstract

The Beyond Reality Image Collection (BRIC) is a set of 648 photos, some painted by an artist and some generated by artificial intelligence. Unlike previous photosets, the BRIC focused on nonrealistic visuals. This collection includes abstract and non-abstract paintings and nonrealistic photographs depicting objects, scenes, animals, humans, and fantastical creatures with varying degrees of unreal elements. We collected evaluative ratings of the photos, using a convenience sample of 16,208 participants in a total of 25,321 sessions. We used multiple evaluation measures: binary positive/negative and like/dislike categorization, seven-point ratings on these attributes, both under no time pressure and under time pressure, and evaluative priming scores. The mean evaluation of the photos on the different measures was highly correlated, but some photos consistently elicited a discrepant evaluative reaction between the measures. The BRIC is a valuable resource for eliciting evaluative reactions and can contribute to research on evaluative processes and affective responses.

**Supplementary Information:**

The online version contains supplementary material available at 10.3758/s13428-024-02586-y.

## Introduction

Photo databases serve as invaluable resources for psychology researchers exploring various facets of human perception, emotion, behavior, evaluation, and cognition (Dal Fabbro et al., 2021; Dawel et al., 2022; Negrão et al., 2021; Popic et al., 2020). These databases provide normalized stimuli, allowing for consistent use in studies, fostering replicability and minimizing confounding variables (Souza et al., 2020). Researchers leverage these databases to investigate domains such as facial recognition (Negrão et al., 2021; Schwartz et al., 2023), object recognition (Brady et al., 2009), emotional processing (Kensinger & Schacter, 2006), emotional perception (Negrão et al., 2021), and memory processes (Semenza, 2009; Kavé et al., 2018). In this article, we introduce a new collection of photos, the Beyond Reality Image Collection (BRIC). The photos in BRIC were generated by image-generating artificial intelligence (AI) models or a human painter with the purpose of presenting unrealistic fictional, complex imageries that vary in valence. The BRIC is available for download (https://osf.io/3upme/) and is free to use for research and any other noncommercial purpose.

A wide variety of stimulus databases are available, designed for numerous research purposes. Some databases contain words (Bradley & Lang, 1999a, b) or audio stimuli (Bradley & Lang, 1999a, b), but most databases in psychology contain a collection of photographs featuring diverse visual stimuli, such as faces, facial expressions, scenes, and objects (Dawel et al., 2022). Some of the most cited photo databases in psychology are the *Open Affective Standardized Image Set* (OASIS; Kurdi et al., 2017), which contains free-to-use photos collected from the Internet, the *Chicago Face Database* (CFD; Ma et al., 2015), comprising professionally photographed faces of adult individuals, and the *International Affective Picture System* (IAPS; Lang et al., 1997), consisting of everyday and rare objects and scenes. These databases were published with normative judgment based on hundreds of raters who judged the stimuli on basic attributes, such as valence and arousal.

The photosets that were collected for studies that involve valence, emotion, or evaluation typically contain only real photos, with real people and objects, depicting realistic scenes. The normative ratings vary on attributes, but not on the judgment process: the raters typically provide their judgment by answering direct questions and have ample time to deliberate on each response. The normative judgment data were not collected with indirect measures, or under nonoptimal conditions such as time pressure.

In the present work, we generated a set of nonrealistic photos. The photos depict unreal imageries, such as abstract paintings, drawings of alien creatures, people merged with animals, robotic faces, houses on tall stilts, human faces with nonhuman features, animals with human facial expressions, and odd variations of famous people. These stimuli are different than the realistic photos used in existing photosets of affective visual stimuli. Our purpose was to create a set of nonrealistic photos that would evoke a wide range of emotions by depicting complex and unusual visual stimuli that are highly unfamiliar and not easily categorized into known semantic and thematic fields.

This collection can enhance the generalizability of research findings by providing a diverse set of stimuli that differ from those in existing photosets. It may also serve research goals that require novel, unfamiliar evaluative photos. For example, one research purpose that we had in mind pertained to discrepancies between the evaluation elicited by a single stimulus different evaluation conditions. Because of their unusual, unrealistic, novel nature, we expected some photos to elicit more positive evaluations when assessed immediately rather than after a delay, and other photos to show the opposite pattern. Such photos could be particularly useful in research exploring evaluation processes in various contexts and conditions. For instance, researchers might investigate whether the unintentional evaluation of a photo aligns more closely with a speeded evaluation than with a delayed evaluation. To address such questions, photos that consistently show discrepancies between speeded and non-speeded evaluations would be invaluable.

Because our focus was on evaluation under different conditions, we did not limit our measures of favorability judgment to self-paced direct rating on a continuous scale. Unlike any previous normative data on existing photosets, we included fast rating (with response deadline), binary rating, and the evaluative priming task (EPT; Fazio et al., 1986)—an indirect measure of evaluation that is based on the evaluative priming effect. The EPT is considered sensitive to unintentional evaluation (Fazio et al., 1986; Bar-Anan & Vianello, 2018), providing normative judgment data that was not collected for any of the previously published valanced photosets. The multiple measures would provide convergent evidence regarding the evaluation elicited by these unusual photos. Further, the different measures might find that some photos, perhaps due to their visual complexity and unusual nature, elicit different favorability estimates under suboptimal conditions, when evaluated unintentionally and quickly than intentionally and slowly. Such information can inform future use of the photos in psychological research, for example, for studies focused on automatic vs. nonautomatic evaluation.

## Method

### Participants

This study was conducted via the Project Implicit research website (http://implicit.harvard.edu/), where the participants voluntarily registered to participate in research and were randomly assigned to the study from a pool of available studies. Unlike most studies on that platform, we allowed for repeated random assignment of the participants to the study. A total of 16,208 participants (62.03% women, *M*_age_ = *34.59, SD*_age_ = *14.77*; US nationals = 61.16%, UK = 5.14%, Canada = 3.89%) provided photo judgment in a total of 25,321 sessions.

### Materials

The BRIC contains 648 photos, 102 of which were painted by an artist (Luiza Schulman), with 50 alien creatures and 52 abstract paintings. The other 546 photos were generated using the AI photo-generation websites Midjourney Inc. (2022) and DALL-E (OpenAI, 2021). The photos can be found at https://osf.io/v49rk/.

In the EPT, the target stimuli were adjective words (positive: *outstanding*, *beautiful*, *magnificent*, *marvelous*, *excellent*, *appealing*, *delightful* and *nice*; negative: *horrible*, *miserable*, *hideous*, *dreadful*, *painful*, *repulsive*, *awful*, and *ugly*).

### Procedure

Participants completed three measures: an evaluative priming task (EPT), a self-report measure, and a speeded self-report measure. There were four self-report response scales, randomly assigned between participants: a binary like/dislike scale, a binary positive/negative scale, a seven-point scale with the responses *extremely dislike, moderately dislike, slightly dislike, neutral, slightly like, moderately like,* and *extremely like*, or the same seven-point scale using positive/negative labels instead of like/dislike. Whereas *positive/negative* are common labels for evaluating photos (Dan-Glauser & Scherer, 2011; Kurdi et al., 2017), we added the *like/dislike* labels to provide rating data with a question that focused on subjective judgment more explicitly.

Each participant rated 72 photos in the binary scale condition or 36 photos in the continuous scale condition (because a continuous scale is more cognitively demanding than a binary scale). These photos were selected randomly in each session, from the complete set of 648 photos. Participants rated each photo twice: once in a speeded self-reported evaluation measure and once in the non-speeded measure. Eight of the photos were included as primes in the EPT, randomly chosen from the 72 or 36 photos that were rated in the self-report questionnaires.

The order of the three measures that each participant completed was selected randomly from four possible orders: [speeded, non-speeded, EPT]; [non-speeded, speeded, EPT]; [EPT, speeded, non-speeded]; [EPT, non-speeded, speeded]. When the EPT appeared before the self-report measures, we started the study with passive viewing task for familiarization with the photos. Participants were asked to observe the eight photos, each of them presented for 500 ms, one after the other. Our goal was to somewhat reduce the difference between the different task order conditions in the ease of processing the photos.

#### EPT

Participants completed three blocks of 64 trials, for a total of 192 trials. In each block, each photo appeared four times before a positive target word and four times before a negative target word. The categorization labels were always *positive* and *negative*. The trial sequence began with a 500 ms fixation, followed by a 200 ms prime photo that was replaced by the target word, which remained on the screen until response. An error response triggered the display of error feedback for 500 ms. A blank screen appeared for 500 ms (intertrial interval) between the trials.

#### Non-speeded self-report

The instructions before the self-report questionnaires were as follows: *In this part of the study, we will show you a few photos, one photo at a time. For each photo, please tell us whether [you like it or dislike it]/[it seems positive or negative to you]. There are no right or wrong answers to this question. We are interested in your genuine judgment.* In the non-speeded questionnaire, we added the following text: *This is not a speeded task. Please take your time before you rate each photo.*

On each trial, participants were asked the following: *Is this photo negative or positive?* Or *Do you dislike or like this photo?* The program allowed participants to respond by selecting their response with the computer mouse or by pressing a number key (both options were available for each question). The question appeared at the top of the screen, the target photo on the right side of the screen, and the response options on the left side of the screen. When the participant chose a response, all the other responses disappeared, while the selected response remained on the screen, with the photo, for 300 ms.

#### Speeded self-report

This measure was very similar to the non-speeded self-report, with the following modifications. Before the questionnaire, we added the instruction text: *This is a speeded task. You will have about one second to rate each photo. So, please, rate the photo as quickly as you can.*

Each trial had a response deadline. The response deadlines were shorter when the scale was binary rather than continuous. We varied the response deadline between trials because people probably vary in how quickly they recruit various evaluative processes. When the response scale was binary the response deadlines were as follows: 1800 ms in trials 1–3, 1500 ms in trials 4–6, 1200 ms in trials 7–9, and 900 ms in trials 10–12. We repeated those deadline durations in six mini-blocks of 12 trials, for a total of 72 trials. When the response scale was continuous, the response deadlines were as follows: 2700 ms for trials 1–3, 2400 ms for trials 4–6, 2100 ms for trials 7–9, and 1800 ms for trials 10–12. We repeated those deadline durations in three mini-blocks of 12 trials, for a total of 36 trials.

In each trial, the color of the screen turned slightly closer to red, in steps of 15% of the maximum response duration (e.g., at the times 180, 360, 540, 720, 900, 1080 ms in a trial with a 1200 ms response deadline). If participants did not respond in time, a large white “PLEASE RESPOND FASTER!” warning appeared for 500 ms.

At the end of the study, after completing the EPT and the two self-report measures, participants were thanked and debriefed.

## Results

### Non-speeded rating

**Fig. 1:**
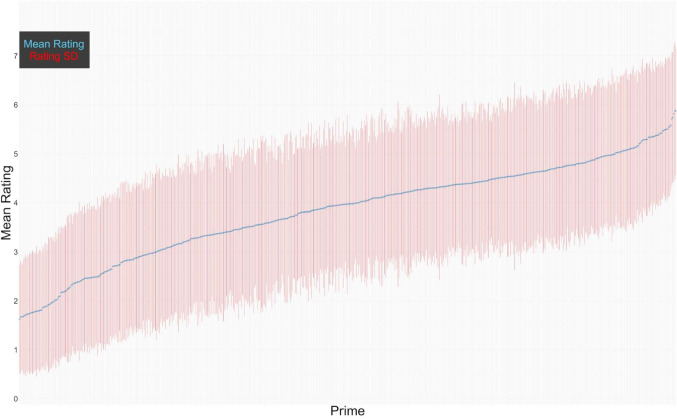
Mean and *SD* of non-speeded continuous scale rating by photo

**Fig. 2:**
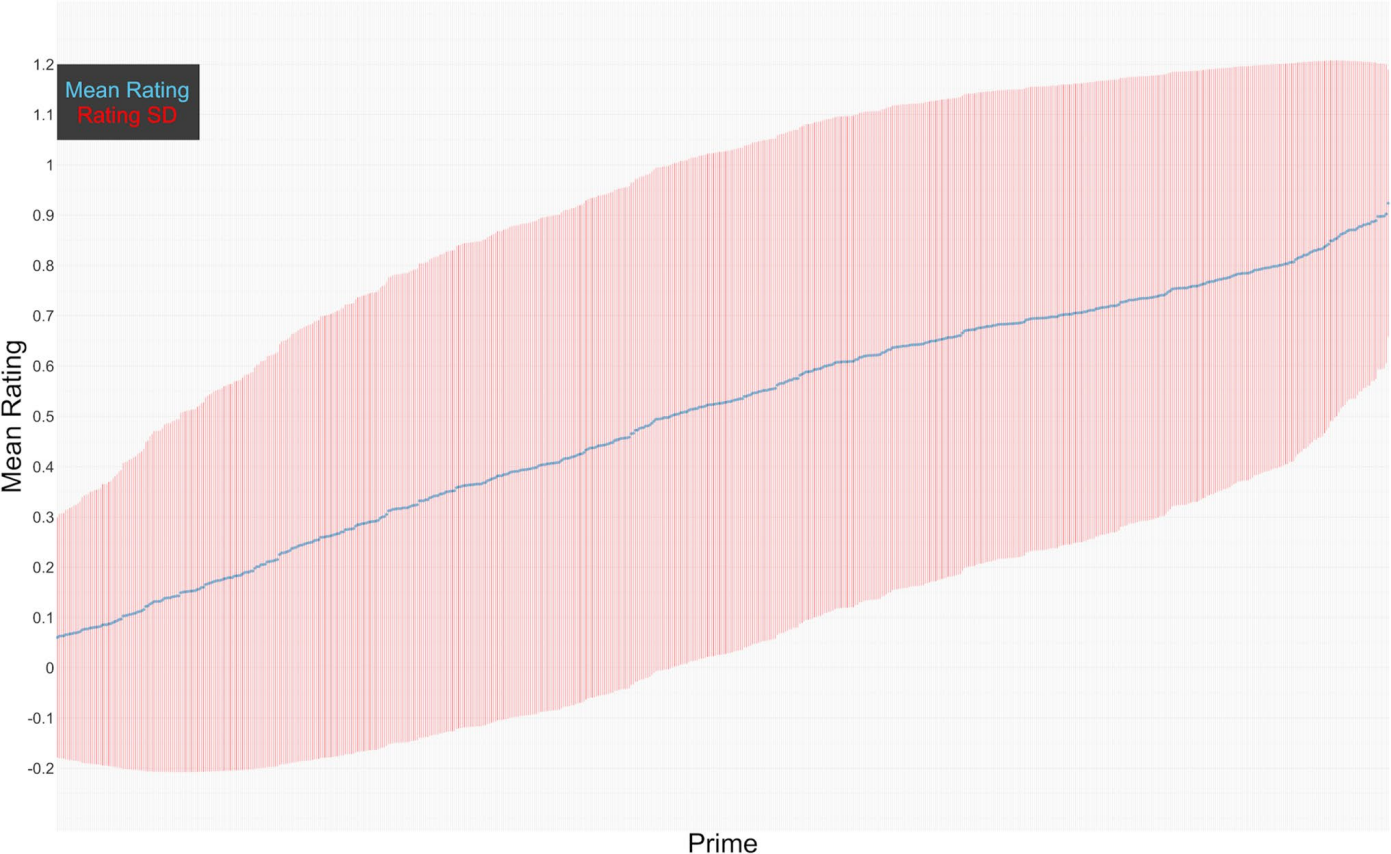
Mean and *SD* of non-speeded rate of positive rating on binary scale by photo

Figure 1 shows the mean non-speeded rating (across response label conditions) on the seven-point scale for each of the photos, with distribution information. Figure 2 shows the rate of positive rating on a binary scale, for each of the photos. Both figures show that there was a wide variability between the photos, with liked, disliked, and neutral photos. On the seven-point rating, 35 (5.4%) of the photos had a mean rating between 1.63 and 2, 101 (15.6%) photos had a mean rating between 2.01 and 3, 199 (30.7%) photos had a mean rating between 3.01 and 4, 253 (39.0%) photos had a mean rating between 4.01 and 5, and 60 (9.3%) photos had a mean rating between 5.01 and 5.88 (the top mean rating). The median rating was 4, the middle of the scale (mean = 3.74, *SD* = 1.15). Just like in the OASIS and the IAPS, the most extreme negative ratings in the BRIC were closer to the negative end of the rating scale than the most extreme positive ratings were to the positive end. On the binary scale, 316 (49%) photos were rated negatively more often than positively.

### Speeded rating

As expected, the shorter the response deadline, the more often it was missed by participants. In the binary response scale, the rates of failure to respond were 4.56%, 4.81%, 9.30%, and 22% for the deadlines 1800, 1500, 1200, and 900 ms, respectively. In the seven-point scale, the rates of failure to respond were 5.25%, 5.20%, 8.35%, and 14.22% for the deadlines 2700, 2400, 2100, and 1800 ms, respectively. As shown in Table 1, the mean response latencies were significantly different as a function of the response deadline, with faster responses observed in trials with shorter response deadlines.

**Table 1:** Response latency by scale and response deadline condition

		Mean		*SD*		25%	Median		75%	
Binary	Non-speeded	1436.49_a_	713.96		942.64			1283.94		1786.44
	1800 ms	757.90_b_	202.26		646.22			760.00		880.17
	1500 ms	708.98_c_	196.06		607.76			713.86		828.90
	1200 ms	679.96_d_	175.64		599.17			697.67		793.44
	900 ms	609.43_e_	139.77		563.01			638.97		696.81
Seven-point	Non-speeded	1754.58_f_	764.83		1281.07			1668.25		2135.90
	2700 ms	1364.67_g_	357.74		1183.67			1400.54		1598.00
	2400 ms	1275.87_h_	359.42		1106.67			1321.56		1515.06
	2100 ms	1236.77_i_	336.87		1088.83			1294.59		1462.03
	1800 ms	1162.11_j_	303.61		1040.67			1225.89		1365.00

*Note*. In the Mean column, different subscripts indicate significant difference

The results suggest that participants provided meaningful ratings, even when they were speeded. As shown in Table 2, the correlation between the mean rating of photos in each of the speeded rating conditions and the two non-speeded rating conditions were above .90, attesting that speeded ratings were far from random. We also computed a separate correlation for each photo (across raters) between each two evaluations of that photo that the participant performed in different measures. As shown in Table 3, the mean of those within-photo correlations was *r* = .70 between the most speeded seven-point rating (1800 ms deadline) and the non-speeded seven-point rating. The equivalent correlation in the binary scale was .57. These reasonable correlations suggest that people often succeeded in providing meaningful ratings even under time pressure. The same tables also show that speeded rating was much more strongly related to non-speeded rating than to the evaluation inferred from performance in the EPT.

**Table 2:** Correlation between the mean rating of photos in the different evaluation measures

		Binary					Seven-point					EPT
		Non-speeded	1800 ms	1500 ms	1200 ms	900 ms	Non-speeded	2700 ms	2400 ms	2100 ms	1800 ms	
Binary	Non-speeded	1	.983	.983	.982	.971	.985	.976	.977	.977	.971	.914
	1800 ms	–	1	.980	.981	.974	.972	.973	.971	.974	.966	.910
	1500 ms	–	–	1	.980	.975	.971	.971	.970	.973	.966	.910
	1200 ms	–	–	–	1	.975	.970	.970	.970	.972	.966	.908
	900 ms	–	–	–	–	1	.958	.963	.958	.964	.959	.896
Seven-point	Non-speeded	–	–	–	–	–	1	.987	.988	.987	.985	.931
	2700 ms	–	–	–	–	–	–	1	.981	.981	.978	.930
	2400 ms	–	–	–	–	–	–	–	1	.980	.978	.927
	2100 ms	–	–	–	–	–	–	–	–	1	.978	.928
	1800 ms	–	–	–	–	–	–	–	–	–	1	.924
EPT		–	–	–	–	–	–	–	–	–	–	1

**Table 3:** Mean correlation between the evaluations (each speeded, non-speeded and EPT score), within each photo

Measure 1	Measure 2	Binary	Seven-point
Fast 900 ms/1800 ms	Slow	.566_cd_	.702_a_
Fast 1200 ms/2100 ms	Slow	.571_c_	.711_a_
Fast 1500 ms/2400 ms	Slow	.579_c_	.700_a_
Fast 1800 ms/2700 ms	Slow	.531_d_	.672_b_
Slow	EPT score	.126_ghi_	.151_e_
Fast 900 ms/1800 ms	EPT score	.115_hi_	.129_fhi_
Fast 1200 ms/2100 ms	EPT score	.116_gh_	.139_efg_
Fast 1500 ms/2400 ms	EPT score	.118_gh_	.145_ef_
Fast 1800 ms/2700 ms	EPT score	.109_i_	.134_efh_

*Note.* Each conditions’ correlation was *z*-transformed using the Fisher transformation. Then, each two conditions were tested with paired *t*-test to examine whether the correlations were significantly different. Correlations that are not significantly different share the same subscript letter

### EPT scores

We computed the EPT evaluation scores following the recommended scoring algorithm (Segal-Gordon et al., 2024; Algorithm #7). Each evaluation score was a *G* score—a scale-invariant non-parametric dominance score (Sriram et al., 2006). To compute *G* evaluation scores, for each participant, for each prime, we first assigned fractional ranks (percentiles) to the *N* latencies of trials that included a stimulus of that prime. We then subtracted 1/2*N* from each fractional rank. We next standardized ranks (i.e., computed the standard normal deviate, with mean = 0 and standard deviation = 1). The evaluation *G* score of each prime was the difference between the mean standardized ranks of trials with positive targets and the mean standardized ranks of trials with negative targets.

**Fig. 3:**
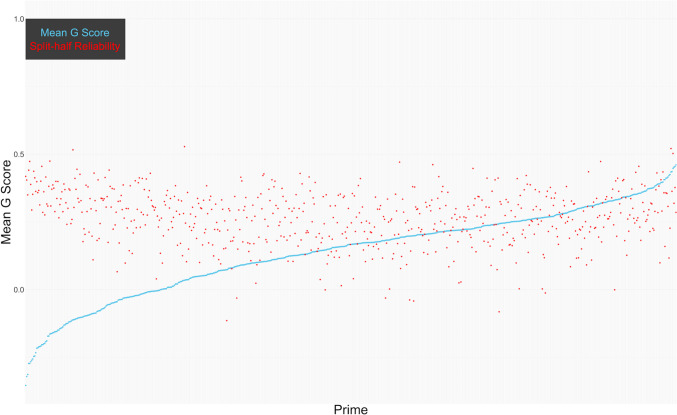
Distribution of the mean EPT evaluation *G* score of each photo and its split-half reliability

Figure 3 shows the mean EPT evaluation *G* score of each photo and its split-half reliability (each half computed from 12 trials). The reliabilities were quite low (range = −.11–.53). This was expected due to the small number of trials for each prime and because the EPT suffers from low internal consistency (Bar-Anan & Nosek, 2014; Gawronski & De Houwer, 2014).

As shown in Table 2, on the aggregate, the estimates of the evaluation score of each photo clearly overcame the reliability problem: the mean EPT evaluation score of each photo was strongly related to the self-reported scores. However, as shown in Table 3, these correlations were much smaller without aggregation, when computed within each individual photo. Table 3 does not provide evidence that the EPT scores are related to speeded rating more than to non-speeded rating. We discuss the theoretical implications of these results in the General Discussion.

### Rating labels

Each participant was assigned to rate the photos using either the labels *dislike/like* or *negative/positive*, while the EPT category labels were always *positive* and *negative*. We computed the mean rating of the 648 photos in each self-report scale, separately for each attribute labels condition. We found high correlation between the mean rating of the 648 photos with the *positive/negative* labels and their rating with the *like/dislike* labels, min = .890, max = .959 (Table S5 in the supplementary online materials [SOM], https://osf.io/e64hc/). When we examined these correlations within each attribute labels pair (e.g., non-speeded binary dislike/like and non-speeded seven-point dislike/like), the correlations were slightly but consistently higher for rating with the *positive/negative* labels than for rating with the *like/dislike* labels (Tables S1–S3 in the SOM).

In contrast, when we examined the correlation within photos (i.e., across participants) between each two measures, all the pairs showed stronger correlations when the attributes were like/dislike than when they were negative/positive rating (Table S4 in the SOM). Despite always using the positive/negative labels in the EPT, even the variability between participants in EPT scores, within of each specific photo, was (on average across the 648 correlations) more strongly related to self-reported evaluation with the like/dislike labels than with the positive/negative labels. This held true for all the correlations between EPT scores and any of the specific self-report measures. In other words, we found that the positive/negative attribute labels are better at measuring individual differences in the valence of photos, whereas the like/dislike attribute labels are better at measuring individual differences in the valence that people assign to each photo.

### Discrepancies

We examined whether some photos elicit consistent discrepancies between different evaluation measures, for example, whether some photos typically elicit a relatively positive evaluation on a seven-point scale and a relatively negative EPT evaluation score. For that purpose, we split the sample randomly into four parts, based on the remainder after dividing the study session ID by 4 (the session ID was created sequentially by Project Implicit’s platform, for every session in each study available on that platform). Next, we computed the mean score of each photo, within each of the four parts, within each of the following five measures: the EPT, speeded-binary, non-speeded-binary, speeded-seven-point, non-speeded-seven-point (we grouped all the deadline conditions together as *speeded*). Next, we ranked those scores, within each of the five measures, within each of the four subsamples. That is, each photo had 20 rank values, one for each measure for each subsample. Next, we computed, for each photo within each subsample, the difference between the photo’s ranks on each pair of measures (i.e., 5*4/2 = 10 pairs). For example, for each photo within each subsample, we computed the difference between the photo’s rank on the EPT evaluation scores and the photo’s rank on the non-speeded seven-point scale. That difference reflected the discrepancy between the two measures in that subsample. At that point, we had 10 discrepancy values for each photo within each subsample. To estimate the consistency of the discrepancies, we first correlated each of the 10 rank difference scores between subsamples. Table 4 shows these 10 correlations for each of the six pairs of subsamples, and their mean correlation. The range of the mean correlations for these 10 correlations was between *r* = .277 and *r* = .485. With 648 observations (the discrepancy score of each photo), all these correlations are statistically significant. This suggests that the discrepancies were not random: higher discrepancy between the evaluation of a photo in two different measures in one subsample predicted higher discrepancy in the evaluation of that photo between these two different measures in a different subsample.

**Table 4:** Discrepancy consistency: Correlations in discrepancy scores for each of the six pairs of subsamples, and the mean of that correlation

Measure 1	Measure 2	1 with 2	1 with 3	1 with 4	2 with 3	2 with 4	3 with 4	Mean
Binary slow	Seven-point slow	.349	.245	.303	.295	.310	.205	.285
Binary slow	Binary fast	.514	.490	.402	.470	.417	.405	.485
Binary slow	Seven-point fast	.419	.256	.293	.242	.312	.292	.303
Binary slow	EPT score	.323	.365	.361	.421	.354	.470	.384
Seven-point slow	Binary fast	.475	.453	.444	.483	.414	.374	.441
Seven-point slow	Seven-point fast	.390	.343	.343	.358	.396	.357	.365
Seven-point slow	EPT score	.290	.370	.329	.408	.326	.417	.357
Binary fast	Seven-point fast	.291	.247	.269	.298	.303	.252	.277
Binary fast	EPT score	.292	.401	.348	.437	.350	.440	.379
Seven-point fast	EPT score	.184	.390	.325	.410	.346	.398	.360

*Note.* The correlations are between the discrepancy scores of the 648 photos in two different subsamples (the entire sample was divided randomly into four subsamples). The discrepancy score of each photo is the difference between the ranking of the mean evaluation score of that photo in Measure 1 and its ranking in Measure 2

The discrepancy information for each photo is posted as a part of the photo database. In Table 5, we show six examples: three photos that were consistently evaluated more positively in the EPT than in the seven-point slow rating, and three photos that were consistently evaluated more negative in the EPT than in the seven-point slow rating.

**Table 5:**
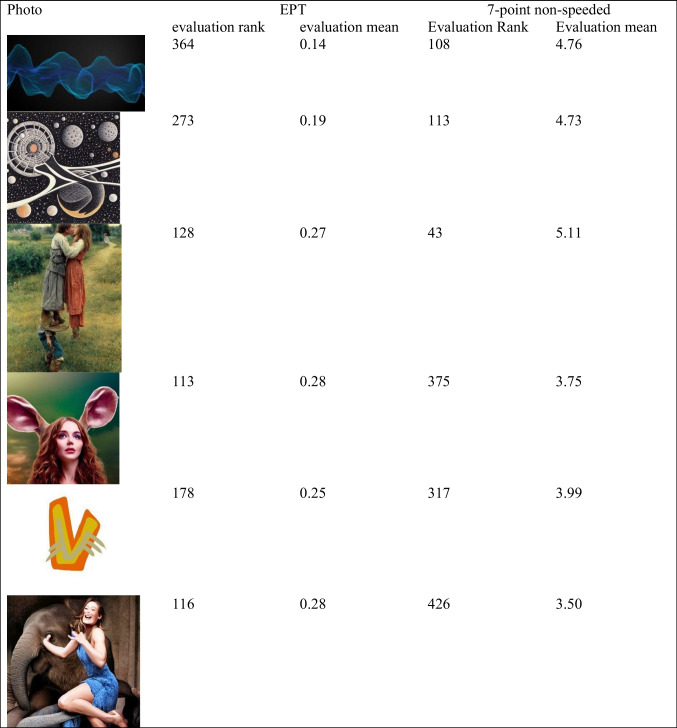
Example photos that elicited discrepant evaluations

*Note*. These photos showed discrepancies between their evaluation score in the EPT and the seven-point non-speeded measure. Lower ranks indicate more favorable evaluations

## Discussion

In this research, we developed the Beyond Reality Image Collection (BRIC) by creating and curating 648 unrealistic photographs and collecting rating norms of people's favorability judgment (evaluation) of those photos. The ratings were provided with and without time pressure, on a binary scale and on a seven-point scale. We also collected evaluative priming scores for each of the photos. The photos and their ratings can be useful in research that requires eliciting evaluative and affective reactions, and in developing evaluation measures.

In comparison to the current frequently used affective image collections, the IAPS (Lang et al., 1997) and OASIS (Kurdi et al., 2017), our collection has slightly fewer photos (*N*s = 648, 704, and 900 in the BRIC, IAPS, and OASIS, respectively). Unlike the IAPS and OASIS, the BRIC includes paintings, abstract drawings, and a wide variety of imagery that is emotionally charged despite depicting unrealistic content. The evaluative norms that we collected are based on a larger number of direct ratings (mean *N* for non-speeded rating of a single photo = 2169, median = 2181, range = 1712–2618 in comparison to *N* = 700, 103 for the IAPS and OASIS, respectively). Like OASIS (but not the IAPS), the BRIC is free to use in any research endeavor and settings (e.g., over the Internet), with no need for registration or permission. Unlike the IAPS and OASIS, we provide norm ratings under time pressure (mean *N* per photo = 1974, median = 1981, range = 1569–2444) and evaluative priming score (mean *N* per photo = 292, median = 292, range = 210–372).

The multiple measures used in the present research provided information about the discrepancy between the evaluative responses that each photo elicits in different measurement contexts. For example, based on the present data, researchers can choose stimulus photos that would elicit different speeded vs. non-speeded evaluative reactions.

The extensive evaluation data that we collected is valuable not only for selecting stimuli for manipulation and measurement procedures but also for investigating evaluative processes. For example, in the present research, we conducted initial analyses on the relationship between evaluative priming and speeded versus non-speeded evaluations. Because unintentional evaluation is generally fast and requires minimal cognitive resources, previous studies have proposed and found that speeded self-reported evaluations align more closely with indirect measures of unintentional evaluation than with non-speeded self-report measures (Ranganath et al., 2008). However, the act of intending to evaluate might trigger processes that, while still undemanding of cognitive resources, differ from those occurring unintentionally when encountering a stimulus. Indeed, subsequent research found opposite results, associating speeded self-reported evaluations with direct measures of deliberate evaluation rather than with indirect measures of unintentional, uncontrolled evaluation (Bar-Anan & Vianello, 2018). In the present research, speeded evaluations were more strongly related to non-speeded evaluation that to EPT score, and EPT scores were not related to speeded evaluations more strongly than to non-speeded evaluations. This result suggests that the evaluative priming effect does not capture evaluative processes that are particularly similar to those that underlie evaluation under time pressure.

Other potentially useful future investigations of the present data may attempt to improve the scoring of the EPT data. This dataset includes more than 183 million trials, and each can be paired with the self-reported evaluation of the prime stimulus. Such data may feed machine learning or computational modeling that may improve upon present knowledge about the most suitable scoring algorithm for EPT data (Segal-Gordon et al., 2024).

Our focus in developing the BRIC was on evaluation. We attempted to create photos that would elicit evaluations that would vary widely on valence. In future research, it would be informative to enrich the normative information with judgment of other attributes. For now, we only conducted a secondary collection of a small sample of arousal ratings of the BRIC (more details on that study in the SOM). The photos elicited a wide distribution of arousal levels: range = 2.78–5.87, on a seven-point scale. Notably, there was a strong negative correlation between the mean continuous evaluation of the photos and the arousal ratings, *r*(648) = −.76, *p* < .0001. Figure S2 shows that relationship in a scatter plot, revealing that the most arousing photos were the most negative photos, whereas the least arousing photos were the relatively neutral photos. The most positive photos were more arousing than neutral photos, but still, not as arousing as the most negative photos. Therefore, the BRIC does not seem useful for research purposes that require positive and negative photos that are equally arousing. Possibly, the unusual nature of these unrealistic photos increased arousal when the content seemed negative but restricted arousal when the content seemed positive. Perhaps lack of realism adds uneasy, eerie feelings to disturbing scenes but detracts from the excitement that beautiful or happy views evoke.

In conclusion, in the present work, we added a set of nonrealistic photos for research on affective and evaluative psychological processes and response. The Beyond Reality Image Collection (BRIC) offers unique and diverse stimuli that can facilitate a deeper understanding of how individuals evaluate and respond to images that deviate from reality. This resource not only complements existing image databases but also opens new avenues for exploring the nuances of evaluative priming, time-pressured judgments, and their underlying cognitive mechanisms.

## Supplementary Information

Below is the link to the electronic supplementary material.

Supplementary file1 (PDF 3.66 MB)

## Data Availability

The datasets generated during and/or analyzed during the current study are available at https://osf.io/3upme/.

## Code availability

The code files for the analysis of the current study are available at https://osf.io/ekvhy/.
