# Supplementary material for: The Beyond Reality Image Collection (BRIC)
Source: Behav Res Methods. 2025 Jan 7;57(1):49. doi: 10.3758/s13428-024-02586-y (PMC11706899; doi:10.3758/s13428-024-02586-y)

Supplemental Online Materials for "The Beyond Reality Image Collection (BRIC)"

Supplement Results

Table S1:

Correlation between the mean rating of photos in each of the speeded rating condition and the two non-speeded rating of like/dislike attributes.

		Binary					7-point					EPT
		**Non-speeded**	**1800 ms**	**1500 ms**	**1200 ms**	**900 ms**	**Non-speeded**	**2700 ms**	**2400 ms**	**2100 ms**	**1800 ms**	
**Binary**	**Non-speeded**	1	.974	.974	.975	.957	.983	.973	.967	.965	.956	.866
	**1800 ms**	-	1	.958	.961	.944	.962	.950	.954	.954	.939	.858
	**1500 ms**	-	-	1	.960	.950	.960	.951	.951	.952	.940	.861
	**1200 ms**	-	-	-	1	.947	.962	.948	.952	.951	.940	.853
	**900 ms**	-	-	-	-	1	.941	.937	.933	.937	.927	.837
**7-point**	**Non-speeded**	-	-	-	-	-	1	.976	.981	.977	.972	.893
	**2700 ms**	-	-	-	-	-	-	1	.962	.957	.952	.890
	**2400 ms**	-	-	-	-	-	-	-	1	.962	.958	.886
	**2100 ms**	-	-	-	-	-	-	-	-	1	.951	.885
	**1800 ms**	-	-	-	-	-	-	-	-	-	1	.879
**EPT**	**G score**	-	-	-	-	-	-	-	-	-	-	1

Table S2:

Correlation between the mean rating of photos in each of the speeded rating condition and the two non-speeded rating of positive/negative attributes.

		Binary					7-point					EPT
		**Non-speeded**	**1800 ms**	**1500 ms**	**1200 ms**	**900 ms**	**Non-speeded**	**2700 ms**	**2400 ms**	**2100 ms**	**1800 ms**	
**Binary**	**Non-speeded**	1	.975	.977	.974	.961	.978	.964	.965	.965	.958	.909
	**1800 ms**	-	1	.968	.970	.963	.961	.959	.957	.961	.955	.900
	**1500 ms**	-	-	1	.970	.960	.961	.955	.958	.960	.954	.903
	**1200 ms**	-	-	-	1	.974	.960	.956	.957	.960	.954	.901
	**900 ms**	-	-	-	-	1	.947	.946	.942	.950	.945	.888
**7-point**	**Non-speeded**	-	-	-	-	-	1	.980	.982	.980	.977	.923
	**2700 ms**	-	-	-	-	-	-	1	.967	.969	.965	.917
	**2400 ms**	-	-	-	-	-	-	-	1	.968	.966	.909
	**2100 ms**	-	-	-	-	-	-	-	-	1	.969	.920
	**1800 ms**	-	-	-	-	-	-	-	-	-	1	.912
**EPT**	**G score**	-	-	-	-	-	-	-	-	-	-	1

Table S3:

The difference between the correlations between the mean rating of photos in each of the speeded rating condition and the two non-speeded rating, between the two attributes; positive/negative and like/dislike.

		**Binary**					**7-point**					**EPT**
		**Non-speeded**	**1800 ms**	**1500 ms**	**1200 ms**	**900 ms**	**Non-speeded**	**2700 ms**	**2400 ms**	**2100 ms**	**1800 ms**	
**Binary**	**Non-speeded**	0	.001	.003	-.001	.003	-.005	.001	-.001	0	.002	.043
	**1800 ms**	-	0	.010	.010	.019	-.001	.009	.003	.007	.016	.042
	**1500 ms**	-	-	0	.011	.010	.001	.004	.007	.008	.014	.042
	**1200 ms**	-	-	-	0	.013	-.002	.008	.006	.008	.014	.047
	**900 ms**	-	-	-	-	0	.006	.008	.009	.013	.018	.051
**7-point**	**Non-speeded**	-	-	-	-	-	0	.004	.001	.003	.005	.030
	**2700 ms**	-	-	-	-	-	-	0	.005	.012	.013	.028
	**2400 ms**	-	-	-	-	-	-	-	0	.006	.008	.023
	**2100 ms**	-	-	-	-	-	-	-	-	0	.019	.034
	**1800 ms**	-	-	-	-	-	-	-	-	-	0	.033
**EPT**	**G score**	-	-	-	-	-	-	-	-	-	-	0

Table S4:

Mean correlation between the evaluations (each speeded, non-speeded and EPT score), within each prime photo, of attributes.

**Rating scale labels**		**Binary**	**Binary**	**Binary**	**7-point**	**7-point**	**7-point**
**Measure 1**	**Measure 2**	**Like/Dislike**	**Positive/Negative**	**Difference**	**Like/Dislike**	**Positive/Negative**	**Difference**
Fast 900ms / 1800ms	Slow	.715	.727	.012	.735	.710	-.025
Fast 1200ms / 2100ms	Slow	.716	.650	-.066	.745	.702	-.043
Fast 1500ms / 2400ms	Slow	.718	.639	-.079	.738	.703	-.035
Fast 1800ms / 2700ms	Slow	.654	.609	-.045	.723	.672	-.050
Slow	EPT score	.131	.122	-.010	.158	.145	-.013
Fast 900ms / 1800ms	EPT score	.136	.098	-.038	.139	.126	-.013
Fast 1200ms / 2100ms	EPT score	.122	.116	-.006	.147	.143	-.005
Fast 1500ms / 2400ms	EPT score	.126	.118	-.008	.153	.141	-.012
Fast 1800ms / 2700ms	EPT score	.118	.095	-.022	.156	.124	-.032

Table S5:

The correlations between the mean rating of photos in each of the speeded rating condition and the two non-speeded rating, between the two attributes; positive/negative and like/dislike.

			**Positive/negative**									
			**Binary**					**7-point**				
			**Non-speeded**	**1800 ms**	**1500 ms**	**1200 ms**	**900 ms**	**Non-speeded**	**2700 ms**	**2400 ms**	**2100 ms**	**1800 ms**
**Like/dislike**	**Binary**	**Non-speeded**	.939	.931	.928	.934	.919	.933	.923	.925	.928	.924
		**1800 ms**	.922	.930	.925	.930	.917	.919	.918	.915	.923	.917
		**1500 ms**	.920	.926	.925	.926	.917	.914	.916	.914	.917	.915
		**1200 ms**	.917	.922	.918	.928	.915	.912	.909	.911	.914	.913
		**900 ms**	.899	.910	.908	.916	.908	.890	.895	.891	.898	.897
	**7-point**	**Non-speeded**	.945	.938	.935	.938	.926	.959	.946	.947	.950	.947
		**2700 ms**	.932	.935	.930	.935	.923	.943	.938	.937	.942	.938
		**2400 ms**	.932	.931	.927	.932	.920	.944	.939	.940	.940	.937
		**2100 ms**	.936	.937	.933	.938	.927	.943	.940	.937	.943	.938
		**1800 ms**	.924	.923	.920	.925	.915	.937	.931	.930	.936	.936

Arousal Ratings

The participants (N = 4,626; 77.93% Female, M_{age} = 32.73; SD_{age} = 14.81) came from the same participant pool as the main study (Project Implicit). We instructed participants as follows:

We will ask you to rate a series of pictures in terms of the amount of emotion that they evoke. In other words, we would like to know how much emotional intensity the picture creates; whether the picture captures something good or bad doesn't matter. We are interested only in the degree of excitement, energy, or intensity of feeling it represents.

Each participant rated 80 photos in two blocks. We instructed participants to ignore The question was "How intense is the emotion that this photo evokes?" along side a reminder to disregard how positive or negative the photo is (wording based on the study that collected arousal norms for the OASIS; Kurdi et al., 2017). The response options were: Extremely low intensity, Moderately low, intensity, Somewhat low intensity, Neutral, Somewhat high, intensity, Moderately high intensity, Extremely high intensity. The ratings were self-paced with no response deadline. The photos were selected randomly for each participant.

The mean number of arousal ratings for a photo was M = 348.60 (SD = 23.94), *Range* = 274 - 427. Figure S1 shows the distribution of the mean arousal ratings across photos (*Range* = 2.78, - 5.87). The correlation between the mean continuous evaluation of the photos and the arousal ratings was $r(648) = -.76$, $p < .0001$. Figure S2 shows that relationship in a scatter plot.

Figure S1

Mean and SD arousal rating by photo.

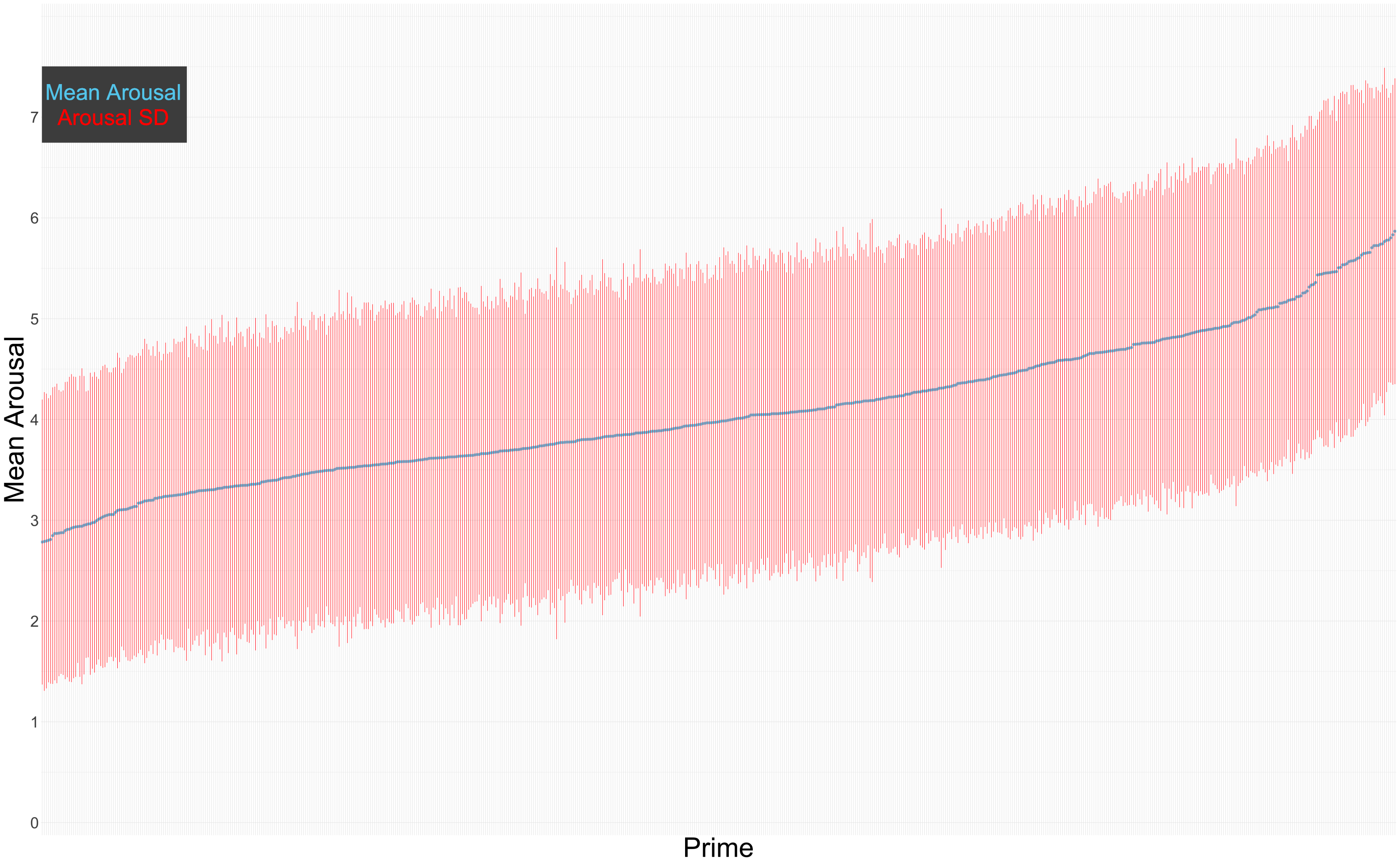

Figure S2

Mean arousal and valance ratings of the photos.

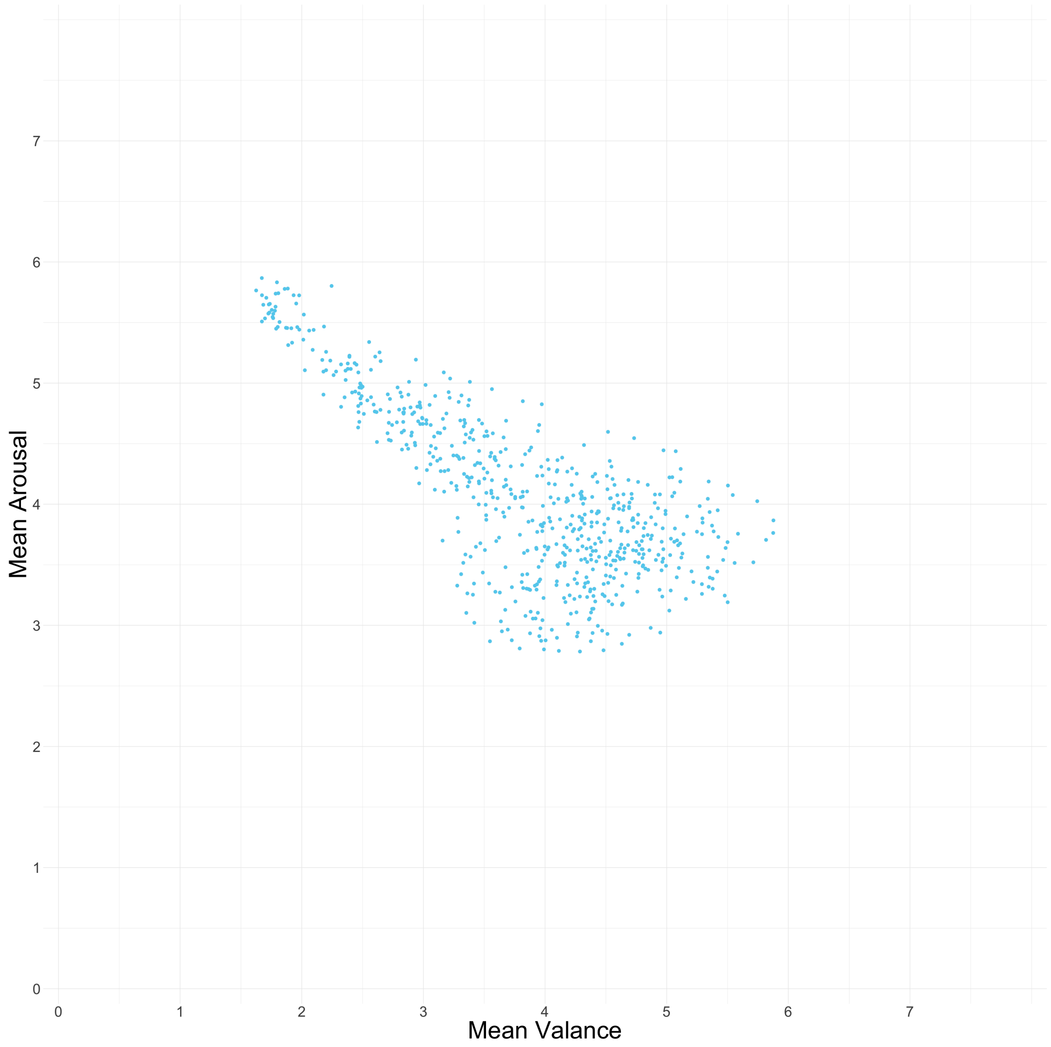

Supplement: Supplementary file 1 — Supplementary file1 (PDF 3.66 MB) [file 13428_2024_2586_MOESM1_ESM.pdf]
